# Conference of Sanitary Inspectors' Association at Buxton: Presidential Address

**Published:** 1922-10

**Authors:** 


### THE SANITARY INSPECTOR.

### CONFERENCE OF SANITARY INSPECTORS' ASSOCIATION AT BUXTON.

IT is a common cry nowadays that we are in danger of being over-inspected, and even that we have passed that interesting stage. In these circumstances it may be interesting to recall that in the fourteenth century a man was hanged for causing a smoke nuisance. Those of us who complain of over-inspection would doubtless, if challenged, concede that the work of the Sanitary Inspector is essential to the life of the community. The public are apt to forget the valuable work accomplished by Sanitary Inspectors throughout the country. But for the Sanitary Inspector, the farmer, too often complacent in his contemplation of a filthy cowshed; the meat or fruit vendor, reluctant to sacrifice an immediate profit; and others of their kind, would very soon relapse into conditions which would tend to restore the appalling infant mortality rate of thirty or forty years ago.

In the Sanitary Inspector we all have a real friend. His calling is one which demands energy, tact, discretion and enthusiasm. Some of us who witnessed the way in which, resisting the delightful surroundings of Buxton, he shut himself up in a hall and threw himself into the absorbing sanitary questions which were discussed at his Conferences, will have no hesitation in testifying to his enthusiasm. It is the more necessary to make it clear that such questions as salaries and conditions of service for these officers are demanding pressing and sympathetic attention. Many of them, it is feared, are seriously underpaid, and this alone is apt to sap enthusiasm.

Sir William Collins, in his presidential address, drew attention to the fact that an important question as affecting the examinations for the position of Sanitary Inspector is at present engaging the attention of the Ministry of Health. Diplomas are granted both by the Royal Sanitary Institute and the Sanitary Inspectors' Examination Board. The Inspectors themselves are represented on the latter Board, as we should expect to find in these twentieth century days, and it seems somewhat anomalous that two separate and distinct methods of examination should prevail. A statement by Sir William Collins, which was loudly applauded, was to the effect that the examiners in any case should not be the teachers of the candidates; this appears to be an unexceptionable proposition. So far as the unification of the separate schemes of examination is concerned, the Ministry may look for public support if they take steps to bring this about.

Sanitary Inspectors are recommended by their President to remain students all their lives, and to keep abreast of the knowledge which bears on the intelligent performance of their duties. We hope in our pages to continue to bring under notice from time to time matters of general interest, both administrative and scientific, in the domain of public health, and that these will contribute to the sources of diversion in which those engaged in sanitary duties, following their president's advice, will find both interest and profit.

### Presidential Address.

Sir William Collins found it difficult to speak without emotion of his immediate predecessor, Sir James Crichton Browne, who had presided over these Conferences since the beginning of the century. He paid high tribute to his eloquent expositions of contemporary sanitary science, and said that they were illuminated with epigrams and adorned with humour. He passed in review the work of his several predecessors—Crichton Browne, Gilzean Reid, John Hutton, Benjamin Ward Richardson, back to Chadwick—and spoke of the debt which the nation owes to them. It had been his good fortune to know them all personally. He described the labours of the first President of the Association, Sir Edwin Chadwick, in the cause of sanitary science, and the opposition which he encountered, and paid tribute to his genius in passages of eloquence which call for those same encomiums which he passed on his predecessors. What were the main conclusions, he continued, at which the first President arrived as the result of his study of how the poor lived and died in mid-nineteenth-century England? What were the practical applications of his great "sanitary idea"? They were, to quote his own words: "That the various forms of epidemic, endemic, and other disease are caused or aggravated, or propagated, chiefly amongst the labouring classes by atmospheric impurities produced by decomposing animal and vegetable substances, by damp and filth, and close and overcrowded dwellings. That such disease, whenever its attacks are frequent, is always found in connection with the physical circumstances above specified, and that when these circumstances are removed by drainage, proper cleansing, better ventilation, and other means of diminishing atmospheric impurity, the frequency and intensity of such disease are abated, and where the removal of the noxious agencies appears to be complete, such disease almost entirely disappears. That cases of smallpox, of typhus, and of others of the ordinary epidemics occur in the greatest proportion in common conditions of foul air, from stagnant putrefaction, from bad house drainage, from sewers of deposit, from excrement-sodden sites, from filthy street surfaces, from impure water, and from overcrowding in private houses and in public institutions. That the entire removal of such conditions by complete sanitation and by improved dwellings is the effectual preventive of diseases of these species, and of ordinary, as well as of extraordinary, epidemic visitations. That when such diseases continue to occur their spread is best prevented by the separation of the unaffected from the affected, by home treatment if possible, if not, by providing small temporary accommodation; in either case obviating the necessity of removing the sick to a distance, and the danger of aggregating epidemic cases in large hospitals, a proceeding liable to augment the death-rates during epidemics."

Chadwick said he had been brought up in the belief that epidemics were largely "climatorial" in causation, but he emphasised that they were largely communicable by infection. He enunciated rules for the sanitary construction of dwellings and hospitals, laying stress on ample cubic space to get rid of "vitiated, phthisis-producing air, and (if the crowding is intense) fever-producing air." He insisted on personal cleanliness as reducing liability to infection, and proclaimed the efficacy of the "active application of soap and water." He saw the need for a radical reform in our local government system, with public representative local authorities, responsible for drainage, water supply and other matters of a communal sanitary nature under unified control.

Chadwick and his co-workers, then, did not seek the causes of pestilence in the visitations of an offended deity, in the convulsions of nature, in obscure telluric disturbances, or in epidemic constitutions of the atmosphere. They regarded zymotic diseases as the nemesis of hygienic short-comings, the evil fruitage of the transgression of sanitary laws, the penalty of physical and moral misdoing or neglect. They taught that the battleground with disease must be without rather than within the body, and that the pathogenic factors are not outside our knowledge or our power, but largely within our comprehension and control. As one of them said, "The human family have now lived in communities more than six thousand years, yet they have not learned to make their habitations clean. When we have mastered that lesson we shall have conquered epidemics." As another declared: "The vigour of their own lives is the best security men have against the invasion of their organisation by low corpuscular forms of life. . . . In unhealthy places, the exclusion of one form of disease is of little advantage; for other priests minister at the altars instead and sacrifice the victims. . . . Man has as much power to prevent as to cure disease. It will be the duty of the Government, the municipal corporations, and all classes of citizens to render the towns of this country and every establishment where large numbers are collected together perfectly adapted to the wants of the human organisation, and compatible with the full enjoyment of health." These sage doctrines of more than two generations ago, at that time ridiculed as the vapourings of a visionary, have become the veriest commonplaces of daily sanitary routine.

Emerson somewhere says that an institution is the prolonged shadow of one man, and it is to Chadwick that English sanitary institutions are largely traceable. His most enthusiastic disciple would not deny that we have learnt much since his day. Bacteriology was in its infancy when Chadwick was in his eighties. When he was superseded by Sir John Simon and his medical department, a "new spirit" was said to be infused into the central health authority at Whitehall. They held that the Chadwick régime, in their "perfectly proper zeal against filth, immensely under-rated the independent importance of the *contagium virum*."

No one would be so rash as to speak disrespectfully of the bacillus, or of the lowlier micrococcus, nor of the labours of bacteriologists and protozoologists, whose laboratory researches have enriched our nomenclature with a formidable vocabulary, adorned our official reports and filled our library shelves. But one has been sometimes inclined to inquire whether it may not be that the microbes have been conceded too exclusive consideration, whether too great regard has not been given to the seed, too little to the soil, and whether we have not sometimes lost sight of the man and his environment amid the luxuriant flora and fauna so assiduously cultivated in the bacteriological laboratory.

The Chief Medical Officer to the Ministry of Health in his most recent annual report asserts "The sound foundation of personal and national health is the body of man; . . . its health and capacity, its resistance is our only sure defence." The lecturer in bacteriology in the University of Leeds in a paper read on February 24 of this year calls attention to the "limitations of bacteriology in its application to public health," and exemplifies these limits in the case of diphtheria, cerebro-spinal meningitis and influenza. In regard to the first of these diseases a leading article in the "British Medical Journal" for December 10, 1921, regards "the general epidemiological problem of diphtheria as unsolved . . . the hopes excited by the first fruits of bacteriological research have been disappointed. . . . The possibility of either a mutation towards or the continuous development of virulence must be further considered," for "there is manifestly some danger of a rigid adhesion to the ritual of bacteriology being accompanied by a neglect of its scientific essence."

Professor Koch, whose name is associated with the tubercle bacillus, and who in his work entitled "The Cure of Consumption," excited hopes in the virtues of tuberculin which have been cruelly disappointed, in a later pronouncement (1901) declared: "It is the overcrowded dwellings of the poor that we have to regard as the true breeding places of tuberculosis; it is out of them that the disease always crops up anew, and it is to the abolition of these conditions that we must first and foremost direct our attention if we wish to attack the evil at its root and to wage war against it with effective weapons."

Take the case of typhus fever, pre-eminently regarded by the older sanitarians as a "filth disease"; no micro-organism has as yet been established as its cause, yet no disease has in the record of our own vital statistics, or in recent experience in Eastern Europe, proved more definitely related to mal-hygiene or more amenable to ordinary sanitary measures and decent conditions of living. You may speak of its hypothetical organism as louse-borne, you may describe it as ultra-visible or a filter-passer, but the disease is nevertheless vanquished by the gospel of soap and water and yields submissively to the Chadwick touch.

I could multiply instances indefinitely to show that sanitation has not been pushed off the stage by the discoveries or the ritual of the laboratory. The broad lines of the teaching of your first President, to whom sanitary science owes an incalculable debt, and to whom you can collectively and individually trace your origin, your method and your practice, have not been superseded and demolished, but rather corroborated and illustrated, by the most recent conclusions of sound clinical and pathological research.

Seeing, then, that the Sanitary Inspector, no longer infelicitously miscalled the Inspector of Nuisances, is the afferent and efferent agent of the local authority in matters of public health, at once its eyes and ears and nose in the detection of disease-causes, and its hands and feet in executing its methods of disease-prevention and control, let us glance at his duties, his qualifications for discharging them, and the status and emoluments of his office.

In the Sanitary Officers' Order of March 28 of this year I find a catalogue of his duties, which if exhaustive by implication is not so by specific enumeration, but is presumably authoritative. These comprise action in regard to noxious trades, water supply, food supplies, infectious diseases, scavenging supervision, diseases of animals, the Rats and Mice (Destruction) Act, the Canal Boats Acts, as well as that which comes upon him daily, the systematic inspection and report on the sanitary circumstances of his district and the carrying out, under the direction of the Medical Officer of Health, of all statutory duties, and orders of the local authority or the Minister of Health.

Another question in which, along with the architect and the engineer, Sanitary Inspectors, like all sanitarians, are deeply concerned is the great housing question. When I was chairman of the London County Council and member and chairman of its Housing Committee I took part, in the years 1897 to 1907, in the active application of the Housing of the Working Classes Act (1890), with which I was very familiar. Since then town-planning Acts have been placed on the Statute Book, and the Government Departments have concerned themselves with housing, if not with uniform success, at any rate more directly than ever before. It is curious to recall that there was no statute dealing with this subject prior to 1851, unless it be one of the 35th of Elizabeth prohibiting the erection of houses in London and Westminster lest those cities should become too extensive for those very spacious times. Yet no matter is more proximately associated with the public health, and none, I venture to say, is more essentially municipal or the legitimate concern of local authorities.

The clearance of slum areas and street and town improvements cannot be carried out by private enterprise alone, and the local circumstances are too varied for the national Government appropriately to undertake the task. If local authorities had before the war used the statutory powers they possessed, we should not have had a house famine of such dimensions as we have witnessed, nor the costly methods undertaken, and then in part abandoned, by the State to meet it.

The best distribution of Governmental duties between the legislature and the municipality is a problem which has been accorded different solutions at different times, and is complicated by the development of such services as those of education and the Poor Law, which have been described as national services locally administered. So also the relation of the State and the municipality to the rights of the individual citizens and to the exercise of private enterprise call for a wise discrimination based upon well-defined principles. It ought not to be necessary to argue that communal necessities like drainage, water-supply, means of transit, markets, ports, fire-prevention, the control of infectious and dangerous mental disease, and the abatement of nuisances should be the concern of the community through a responsible and representative local authority. We have during the years of war been living under a régime of nationalisation; the current is now the other way, and de-nationalisation is the vogue. In matters which are not communal in their nature, and in times of peace, the public will rightly resent either a State or a municipal bureaucracy. John Stuart Mill's essay "On Liberty" is still well worth reading; in it he contended that "Each is the proper guardian of his own health, whether bodily, mental or spiritual," and "Over himself, over his own body and mind, the individual is sovereign." Yet Mill, like Herbert Spencer, who waxed warm in vindicating "The Man *versus* the State," was at one with the great individualist in recognising "the law of equal liberties," which ordains that the liberty of each is limited by the observance of like liberty for others. If with Montesquieu we seek for the spirit of our laws, we may find in that aphorism a sound basis on which to build our sanitary legislation and administration, whose end and object the *salus populi* is proverbially the *suprema lex*.

Mill, in the later chapters of his famous essay, deals with the application of "the law of equal liberties" and its translation into practice. He refers to our pharmacy and poisons legislation, to the opium traffic, the sale of alcohol and to slavery. In regard to the latter he significantly observes that "the principle of freedom cannot require that a man should be free not to be free," since "by selling himself as a slave he abdicates his liberty," and this argument, he adds, is capable of wide application. Thus in the case of the sale of noxious drugs which enslave or paralyse the will, we who assisted in drafting the International Opium Convention at the Hague in 1912, bore this principle in mind. We sought the limitation of self-will in the interests of free-will and self-control. The reciprocal influence of physical welfare and mental and moral sanity, in the fullest sense, was a favourite theme of the earlier hygienists, but I refrain from entering upon that theme on this occasion.

The Sanitary Inspector, as the executive officer of local government in matters of health, and in close touch with the public, is bound to keep himself informed on current topics relevant to sanitation in its broadest application. However thorough and satisfactory your preliminary and qualifying examinations may become, it is well, like members of other professions, to remain students all your lives and keep abreast of the knowledge which bears on the intelligent performance of your duties.

I think it was Jowett who advised the employment of leisure in studying, from the speculative side, the profession or business in which one is practically engaged. This should prove an unfailing source of diversion to those engaged in sanitary duties, seeing that the science of healthy living is rich in speculation, and new hypotheses are propounded in the lay and medical Press with almost rhythmical periodicity.

Probably legislatures and municipal bodies were never more ready than they are to-day to listen to the latest word of science, if only they can be assured with no uncertain voice what the latest word of science is, and they are not unmindful that the latest word of science is not invariably the last, and that those who know most are not always those who know best.

